# Ecological and socio-cultural factors influencing *in situ *conservation of crop diversity by traditional Andean households in Peru

**DOI:** 10.1186/1746-4269-7-40

**Published:** 2011-12-06

**Authors:** Dora Velásquez-Milla, Alejandro Casas, Juan Torres-Guevara, Aldo Cruz-Soriano

**Affiliations:** 1 Coordinadora de Ciencia y Tecnología en los Andes (CCTA), Camilo Carrillo 300-A, Lima, Perú; 2 Centro de Investigaciones en Ecosistemas (CIECO). Universidad Nacional Autónoma de México, Campus Morelia, Michoacán, México; 3 Departamento de Biología de la Universidad Nacional Agraria La Molina (UNALM), Av. Universidad s/n, La Molina, Lima, Perú

**Keywords:** Andean native tubers, Andean traditional agriculture, genetic resources, *in situ *conservation, traditional ecological knowledge, traditional crop varieties

## Abstract

**Background:**

The Peruvian Andean region is a main center of plant domestication of the world. There, several tuber species were domesticated and the area lodges one of the most important reservoirs of their varieties and wild relatives. It is also the setting of traditional cultures using and conserving them. However, crop genetic erosion has been reported in the region since several decades ago; therefore, understanding factors influencing both loss and maintenance of crop variation is relevant to design conservation policies. Previous researches have examined factors influencing agrobiodiversity conservation in the region but additional case studies are recognized to be still necessary for a deeper understanding of causes of genetic erosion and for policy design to prevent and remedy it. Our study focused on analyzing (1) variation in richness of traditional varieties of tubers cultivated among households, (2) changes in varieties richness occurred in four consecutive agricultural cycles, and (3) ecological, social, and cultural factors influencing loss and conservation of varieties.

**Methods:**

Richness of farmer varieties of tuber species cultivated by 28 peasant households was monitored in communities of Cajamarca and Huánuco, Peru during four consecutive agricultural cycles (from 2001 to 2005). In-depth interviews were conducted with 12 of the households with higher reputation as conservationists, in order to document farmers' perception of tubers qualities in ecological, social, economic, technological and culinary aspects and how these influence their decisions of conservation priorities. Traditional varieties were identified according to their local names, which were then confronted among farmers and with scientific catalogues in order to identify synonyms. Based on the information documented, indexes of ecological and socio-cultural factors affecting agricultural practices were designed, and their linear correlations and multivariate relations with varieties richness managed per household were analyzed in order to explore factors with higher influence on conservation of crop variation.

**Results:**

A total of 1483 and 507 farmer varieties of tuber species were found in the whole sample and period studied in Huánuco and Cajamarca, respectively. Significantly more varieties managed per household per year were recorded in Huánuco (146.39 ± 12.02) than in Cajamarca (44.55 ± 9.26), and marked differences in number of varieties per year were documented among households within each region (78.25 to 246.50 in Huánuco, 7.50 to 144.00 in Cajamarca). Correlation and multivariate analyses identified that the extent of agricultural area managed by households, cultural identity, practicing of traditional agricultural techniques, and level of self-sufficiency, are meaningful factors influencing higher varieties richness maintained by households. Yield and culinary attributes were considered by people as main features for selecting and deciding which varieties are priorities for conservation.

**Conclusions:**

Maintenance and promotion of indigenous Andean culture is crucial for ensuring conservation of both traditional agroecological systems and agrobiodiversity. Policies supporting Andean culture (through educational, cultural and economic programs) are therefore directly connected with conservation of traditional farmer varieties. Promotion of seed availability and interchange are effective actions for maintaining and developing diversity, but using and valuing native tubers at regional, national and international levels are fundamental motivations to enhance policies and processes in this direction.

## Background

The Andean region of Peru is one of the main centers of domestication and diversification of crop plants of the world [1-3]. The great genetic variation of crop species occurring in the area resulted from evolutionary processes influenced by the high environmental variety of local mountain ecosystems, as well as by processes of domestication conducted by native cultures that have modeled crops according to their also variable and changing cosmovision, knowledge and technology throughout history [4-7].

**Figure 1:**
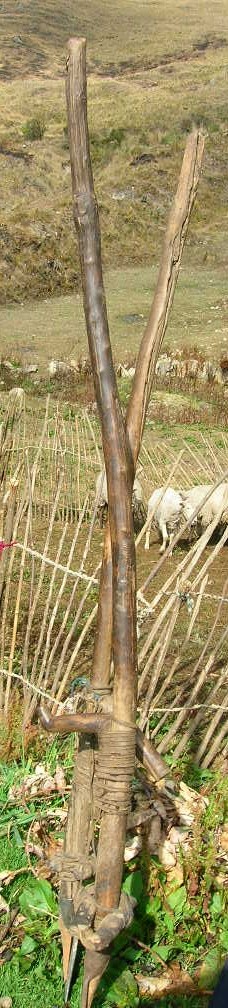
**The *chakitaqlla*, a stick traditionally used by Andean farmers for cultivating native tubers**. The photo (by Dora Velásquez, 2008) is from the village of Monte Azul, Kichki, Huánuco, in Sabino Alejo's farm.

The current Andean traditional agriculture derived from a "classic indigenous agriculture" [8] practiced in pre-Columbian times, characterized by numerous strategies of risk management [8-10] which can be still observed among indigenous communities across the Peruvian mountains [11]. The "agriculture of Andean native tubers", as defined by Morlon [12] is a highly specialized production system developed in valleys and slopes at elevations between 3300 and 4200 m. This system is organized to produce native tubers including seven potato species (*Solanum ajanhuiri, S. chaucha, S. curtilobum*, *S. juzepzuckii*, *S. phureja*, *S. stenotomum*, and *S. tuberosum *[13]), with about 3000 varieties characterized by botanical descriptors [2], "oca" (*Oxalis tuberosa*) with at least 50 technically described varieties, "olluco" (*Ullucus tuberosus*) with 50 to 70 clones, and "mashua" (*Tropaeolum tuberosum*) with nearly 100 described varieties [14]. This agricultural system is carried out in one of the most difficult settings for practicing plant cultivation of the world, given the elevations, frosts, pronounced slopes and soil types characterizing the highland Andean environments [12,15]. The system involves short cultivation periods alternating with large fallow periods, typically using the tilling tool called ***chakitaqlla ***in Quechua (Figure 1), which is adapted to manage the particular soil types of the region [12].

But, however the importance of native tubers for highland Andean peasants culture, signs of genetic erosion at both species and intra-specific levels have been documented in several regions. In one of the earliest studies, Ochoa [16] documented cases of genetic erosion of potato in Chile, Bolivia, and Peru. Later on, in Peru Hawkes [17] and Franco [18] documented loss of native varieties of *S. stenotomum *subsp. *stenotomum*, and *S. s*. subsp. *goniocalyx *in Cusco, as well as loss of native potatoes in Ancash and potato wild relatives in Cusco, Apurímac and Lima. This process appears to have been especially drastic during the last three decades. For instance, Figueroa [19] documented that in Warmiragra, Huánuco, a progressive and accelerated trend of decreasing potato variation started in the 1980s.

During the 1990s, important ethnoecological and biogeographic researches by Brush [4-6] and Zimmerer [7,15] among other scholars, allowed identifying ecological and cultural factors influencing decisions of households for maintaining diversity of tubers, especially potatoes. Those studies tested important hypotheses in relation to the specific adaptations of farmer varieties to particular environments, agricultural intensification as cause of genetic erosion, and the importance of traditional networks of seed exchange in agrobiodiversity conservation [4-7,15]. However, both new and old questions and hypotheses remain to be answered and tested and require more research and case studies before new changing contexts [5].

By the end of the 1980s and early 1990s several initiatives for *in situ *conservation of agrobiodiversity were promoted in Peru by governmental and non-governmental organizations and academic groups. These initiatives then crystallized in the project "*In situ *conservation of native crops and their wild relatives in Peru" (which ahead will be shortly called "*in situ *project"). This project was conducted from 2001 to 2006, and became one of the most important Peruvian efforts of *in situ *conservation of agrobiodiversity. It was an inter-institutional program documenting and promoting conservation of agrobiodiversity of Andean and Amazonian regions of Peru. The "*in situ *project" recorded nearly 9000 names used by local peoples for designating native variants of several crop species of tubers [20-23]. We based our study on this type of varieties, which were considered as units of variation recognized and named by the traditional agriculturalists. Ahead in the text these varieties will be called "traditional farmer varieties" and we consider them analogous to those called "farmer varieties" by Zimmerer [15].

By monitoring numerous production units during the agricultural cycles from 2001 to 2005, the "*in situ *project" documented differences in richness of traditional farmer varieties among households and regions, and identified that some varieties remained constant in parcels (regionally called ***chacras***) whereas others changed from cycle to cycle.

The research effort of the "*in situ *project" resulted in a great amount of information that has been only partially used for *in situ *conservation programs and public policies but that potentially may have more substantial contribution. Some institutions and scholars have made attempts to systematize and analyze the information but there is still much to do, especially for identifying the main factors influencing loss, maintenance, and generation of varieties richness by households. This latter task is particularly important for designing strategies of *in situ *conservation, and our study aspires to contribute in this direction. Based on information generated by the "*in situ *project", the main questions guiding our current research were: How different the traditional farmer varieties richness is among regions and households?, what factors influence such differences?, what factors determine that certain traditional varieties remain constant (are "not replaceable") whereas others are more commonly lost or substituted by others (are "replaceable")?

We hypothesized that: (1) differences in traditional farmer varieties richness are associated to ecological, technological and socioeconomic variations of the contexts where agriculture takes place; particularly, we supposed that aspects such as the range of environments in which households practice agriculture, the weight of traditional culture influencing strategies of risk management and use of traditional technologies, as well as the degree of dependence of their subsistence on their own agricultural products should be meaningful factors determining in direct proportion higher crop variation; (2) traditional varieties would be "not replaceable" or "replaceable" depending on their role in households subsistence, and their performance in particular environments, technological and cultural contexts of production. Our study aimed at exploring these hypotheses in a sample of households from Huánuco and Cajamarca, looking for identifying factors influencing both loss and conservation of these plant genetic resources.

Exploring these hypotheses was also considered useful for constructing methods to analyze the great amount of data generated by the "*in situ *project". We aspire to contribute to make more relevant use of the information obtained by the "*in situ *project" to design strategies and policies of *in situ *conservation of genetic resources.

## Methods

### Study area

**Figure 2:**
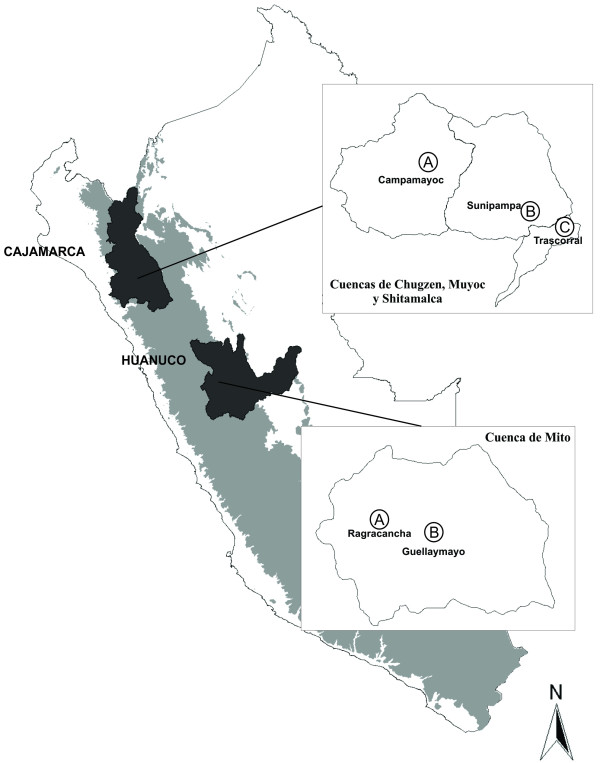
**Study area location**. Northern Sierra (Cajamarca). A) Chugzen Watershed, B) Muyoc Watershed, C) Shitamalca Micro-watershed. Central Sierra (Huánuco): Mito Watershed: A) Ragracancha Micro-watershed, B) Guellaymayo Micro-watershed.

Our study was conducted in the highest elevation zone of mountain agroecosystems of Cajamarca (Northern Sierra region) and Huánuco (Central Sierra region), Peru. In Cajamarca our study covered elevations from 3100 to 4150 m in the watersheds of Chugzen, Muyoc and Shitamalca, Province of San Marcos. In Huánuco our study covered elevations from 3100 to 4300 m in the watershed of Mito, Province of Huánuco (Figure 2). Both study areas have ecological characteristics of Suni and Puna (the later environment also called Jalca in Cajamarca) altitudinal floors [24], with dry seasons from April to October and rainy seasons from November to March, recurrent droughts, and frosts [25]. Cajamarca has higher average temperature and lower annual precipitation (13.1°C and 704 mm, respectively) than Huánuco (9.1°C and 967.3 mm, respectively) [25]. Natural ecosystems are mainly grass steppes but these are strongly degraded.

People of the study area inhabit small and dispersed settlements of difficult access. Most of the population live in poverty and extreme poverty conditions (in 2001, nearly 77.4% and 50.8% in Cajamarca, and 78.9% and 61.9% in Huánuco, respectively), with high levels of illiteracy and strong migration to cities [26,27]. Household economy mainly depends on agriculture oriented to direct consumption of products.

### Evaluation of variation of native tubers and their wild relatives

Traditional farmer varieties of potato, oca, olluco and mashua, recognized and named by farmers, were annually recorded during four sequential agricultural cycles (from 2001 to 2005), in parcels cultivated by a sample of 28 peasant households. Traditional farmer varieties are units of variation recognized and named by local people based on multiple morphological, physiological, phenological, and ecological features, as well as technological and culinary attributes (texture, flavor, color, storing resistance, particular forms of preparation, among others). Traditional farmer varieties are not equivalent to varieties characterized by botanists and agronomists through standardized morphotype descriptors, e.g. [28], neither to those identified through isozyme analysis or neutral genetic markers, e. g. [29,30].

Further in-depth studies were conducted with 12 households (6 from Cajamarca and 6 from Huánuco), which were selected based on their reputation as crop diversity conservationists according to the high number of local varieties they manage, as documented by the Centro IDEAS [31] and IDMA [32]. Therefore, this biased sample of households represents the most favorable conditions for conservation but not the general conditions of crop diversity conservation in the communities studied.

Traditional farmer varieties of tuber crop species were recorded every agricultural cycle during the study period. After obtaining name inventories and photographic material of traditional farmer varieties, a careful process of nomenclature depuration was conducted through workshops in order to corroborate the varieties identity and to identify synonyms. Such process was conducted with people of the households studied, consulting to them doubts about the varieties identity by confronting plant materials and images of different tubers with similar names and similar tubers receiving different names. The process was supported with complementary reviews of common names registered in the most complete database of potato landraces collection available for Peru [33]. Also, it was based on the passport data of oca, olluco and mashua collections [34-36] of the Potato International Center (CIP) genebank. In addition, information from reviews of common names of potato and oca landraces registered in Huánuco by previous studies [37,38] was also taken into account.

Variation of crops managed by the peasant households studied was determined calculating the richness of traditional farmer varieties recognized at household and regional levels per agricultural cycle and for the whole period studied. Taxa identified as wild relatives of potatoes, oca, olluco and mashua were inventoried in all study sites.

### Analysis of factors influencing traditional crop varieties richness

An integrated multi-factor approach to analyze motives of management and maintenance of diversity of varieties in farmers' fields was conducted, inspired in ideas by Zimmerer [7,15] and Brush [5]. Ecological, technological, cultural, and socio-economic aspects of the 12 households more deeply studied were documented through direct observation, surveys and semi-structured interviews, as explained below. The information was analyzed through qualitative and quantitative approaches. For quantitative approaches, in some cases the information obtained was used to construct indexes integrating information related to particular topics (Table 1) and analyzed through generalized linear models and multivariate statistical analyses in order to explore the weight of the factors analyzed in relation to traditional varieties richness managed per household.

**Table 1:** Indexes constructed for integrating information of the study, definitions, and scales of values

INDEX	DEFINITION	SCALE OF VALUES
**INDEX OF PARCEL DISTRIBUTION (IPD) = 0.45 × NPHEZ + 0.35 × NPIEZ + 0.2 × NPZB** - NPHEZ = Number of Parcels of High Elevation Zone (3800 to > 4100 m) - NPIEZ = Number of Parcels of the Intermediate Elevation Zone (3400 to < 3800 m) - NPLEZ = Number of Parcels of Lower Elevation Zoones (3000 to < 3400 m)		

**INDEX OF CULTURAL IDENTITY (ICI) = 0.4 × AAT + 0.25 × QL + 0.2 × AIA + 0.1 × SFA + 0.05 × SChA**		1.0 = Total identity
	
- ***AAT = Appropriation of Agricultural Traditions***	Indicates the number of traditions associated to agriculture (customs, rituals, festivities) recognized, practiced, and transmited by a household head in relation to all traditions associated to agriculture identified in our study.	0.8 to < 1.0 = Very strong identity
	
- ***QL = Quechua Language***	Indicates the degree of use of the Quechua language by the household: 1 = only Quechua, 0.5 = bilingual (Sapnish-Quechua), y 0 = only Spanish.	0.6 to < 0. 8 = Strong identity
	
- ***AIA = Age as Independent Agriculturalist***	Indicates the inverse relation between age at which households' heads started to cultivate native tubers as an independent and the minimum age identified amonh households heads studied.	0.4 to < 0.6 = Intermediate identity
	
- ***SFA = Stimuli of Father as Agriculturalist***	Indicates the age at which the household's head learned agriculture (1 = child, 0.5 = teen ager, 0.25 = adult), the proportion of varieties inherited in relation to all varieties documented, the proportion of land area inherited in relation to the maximum area identified, and origin of land cultivated as independent agriculturalist: 1 = inherited; 0.75 = repartición; 0.50 = renting, an 0.25 = buying.	0.2 to < 0.4 = Weak identity
	
- ***SChA = Stimuli of Children as Agriculturalists***	Indicates the traditional ways (knowledge, technology, mutual help, seeds, land) that consider necessary to give to children to continue withtraditional agriculture of native tubers, minus the modern ways (education, fertilizer, insecticides) also mentioned.	0 to < 0.2 = Very weak identity

**INDEX OF TRADITIONAL AGRICULTURAL MANAGEMENT (ITAM) = 0.75 × SiCAgT + 0.25 × SiCFSm**		1.0 = Total traditional management
	
- ***CTA = Conservation of Traditional Agriculture*** $\text{CTA}=\frac{\text{ACNT}+\text{TT}+\text{MH}}{3}$	- ***ACNT = Area Cultivated with Native Tubers***, which is the area cultivated with native tubers per agriculturalist in relation to the largest area cultivated by the agriculturalists documented.	0.80 a < 1,00 = Very strong traditional management 0.60 a < 0.80 = Strong traditional management 0.40 a < 0.60 = Intermediate traditional management 0.20 a < 0.40 = Weak traditional management 0 a < 0.20 = Very weak traditional management
	
	- ***TT = Traditional Technologies***, which is a relation of key traditional technologies: (i) proportion of land use and fallow, (ii) tilling system (1 = ***raway***, 0.5 = ***pampay***, and 0.25 = furrowing), (iii) proportion of dung bags used in relation to the average used per agriculturalist, and (iv) traditional seed stroring (1 = ***coyona ***or ***saway ***-piles of tubers covered with straw-; 0 = platforms/nets/sacks).	
	
	- ***MH = Mutual Help ***1 = only mutual help, 0.5 = mutual help and payed laborers, y 0.33 = low mutual help and high payed laborers.	
	
- ***CSF = Conservation of Seed Flow*** $\text{CSF}=\frac{\text{SE}+\text{SOM}}{2}$	- ***SE = Search Effort***, the search effort of each agriculturalist relation to the maximum effort identified, calculated as the product of thesearch area: 1 = only local; 2 = only regional; 3 = only inter-regional; 4 = local/regional; 5 = local/inter-regional; 6 = regional/interregional; 7 = local/regional/inter-regional, and the search intensity as the summatory of the number of places visited: No. local places + 2 × No. regional places + 3 × No. in-terregional places.	
	
	- ***SOM = Seed Obtaining Mechanisms***, indicating presence or absence of traditional mechanisms: 1 = barter and present; 0.8 = barter or present; 0.6 = barter, present and buying; 0.4 = buying, barter or present; 0.2 = buying; 0 = none.	

$INDEX\;OF\;SELF-SUFFICIENCY\;\left(ISS\right)=\frac{VPDC}{VPDC+TI}$		1.0 = Total self-sufficiency
	
- **VPDC = Value of Production for Direct Consumption**	Calculated as the monetary value of agricultural production destined to direct consumption by households.	0.80 a < 1.00 = Very high self-sufficiency 0.60 a < 0.80 = High self-sufficiency 0.40 a < 0.60 = Intermediate self-sufficiency
	
- **TI = Total Incomes**	Calculated as the summatory of monetary value of total agricultural production, salaries and incomes derived of social assistance programs.	0.20 a < 0.40 = Low self-sufficiency 0 a > 0.20 = Very low self-sufficiency

$INDEX\;OF\;FAMILY\;SIZE\;\left(IFS\right)=\frac{N^{\circ }\;members\;of\;the\;household}{Maximum\;N^{\circ }\;members\;of\;all\;households\;studied}$		0.80 a 1.00 (9-11 members) = Very numerous 0.50 a < 0.80 (6-8 members) = Numerous 0.30 a < 0.50 (4-5 members) = Intermediate 0.10 a < 0.30 (1-3 members) = Small

$INDEX\;OF\;FAMILY\;SIZE\;\left(IFS\right)=\frac{N^{\circ }\;members\;of\;the\;household}{Maximum\;N^{\circ }\;members\;of\;all\;households\;studied}$		0.80 a 1.00 = Very high 0.60 a < 0.80 = High 0.40 a < 0.60 = Intermediate 0.20 a < 0.40 = Low

#### Environmental factors

Information on environmental conditions (soil types, slope degree, average rainfall and temperature, frost occurrence, vegetation and dominant components in the surrounding area, access to irrigation, among other topics) in which peasants cultivate native tubers and on the spatial organization of parcels (elevation, area, and number of plots) were recorded in the field. People's perception about threats associated to climate (frosts and amount of rain), pests, availability of soil and water and their management solutions was documented through interviews. We also explored people's perception on the presence of wild relatives in the surrounding areas of crop fields and their relation to crops. Influence of spatial distribution of parcels was analyzed by recording the traditional varieties richness in relation to the total surface of parcels that each farmer dedicated to cultivation of tubers. We considered the distribution of parcels in three altitudinal floors (3000-3400 m, 3400-3800 m, and 3800-4100 m, see the index of plots distribution IPD in Tables 1 and 2), which are agroecological zones meaningful in the spatial distribution of particular species and varieties of tubers in the central Peruvian Andes according to studies in the Mantaro Valley [39].

**Table 2:** Ecological, technological and socio-economic factors analyzed and their correlation values (r) with richness of farmer varieties.

	Cajamarca					Huánuco					Both regions				
	
Factor (independent variable)	Agricultural cycle					Agricultural cycle					Agricultural cycle				
	
	2001-2	2002-3	2003-4	2004-5	All years	2001-2	2002-3	2003-4	2004-5	All years	2001-2	2002-3	2003-4	2004-5	All years
Total farms number	-0.093	-0.108	-0.143	-0.035	-0.026	-0.146	-0.146	-0.240	-0.239	-0.249	-0.018	-0.024	0.036	0.089	0.077

Farm Extension	0.131	0.519	0.735*	0.616*	0.443	0.175	0.174	0.234	0.252	0.200	0.164	0.396*	0.433*	0.457**	0.302*

Parcels Number	0.480	0.866**	0.962**	0.926**	0.840**	0.342	0.349	0.521	0.428	0.460	0.169	-0.003	-0.056	-0.042	0.143

Native tubers surface 2001-2005	0.218	0.485	0.603*	0.683*	0.558	0.476	0.473	0.399	0.323	0.308	0.440*	0.684**	0.702**	0.684**	0.614**

Index of parcels distribution (IPD)	0.607*	0.883**	0.838**	0.829**	0.884**	0.071	0.075	0.362	0.447	0.408	0.260	0.011	-0.066	-0.0555	0.169

Index of cultural identity (ICI)	0.113	0.432	0.598*	0.702*	0.483	-0.170	-0.173	-0.244	-0.181	-0.203	0.174	0.444*	0.451*	0.311*	0.166

Index of traditional agricultural management (ITAM)	-0.016	0.314	0.580*	0.662*	0.360	-0.066	-0.061	0.245	0.301	0.293	0.222	0.479**	0.675**	0.681**	0.540**

Index of self-sufficiency (ISS)	0.237	0.226	0.072	0.376	0.374	-0.149	-0.148	-0.245	-0.232	-0.239	0.273*	0.457**	0.490**	0.403*	0.283*

Index of family size (IFS)	0.353	0.674*	0.706*	0.862**	0.658*	0.022	0.021	-0.183	-0.248	-0.238	-0.012	-0.067	-0.033	0.028	0.104

Index of household labor hand (ILH)	0.703*	0.629*	0.241	0.580*	0.670*	-0.031	-0.032	-0.174	-0.242	-0.224	0.051	-0.092	-0.098	-0.071	0.034

*indicates significant correlations with 0.01 < p < 0.05; ** indicates significant correlation with p < 0.01.

#### Cultural factors

We analyzed aspects related to household cultural identity defined according to Quezada [40] as a sense of belonging to the community where people were born. We based our analysis on information obtained through interviews to farmer heads of the households sampled on: (i) the use of the Quechua language, (ii) the practice of traditional rituals and customs associated to agriculture, (iii) the age at which the farmers interviewed learned agricultural practices, (iv) the age at which they became independent agriculturalists, and (v) activities practiced to enhance practice of native tubers agriculture by children. Because of the complexity of the aspects involved, we constructed an index of cultural identity (ICI, see Tables 1 and 2).

In order to determine the factors influencing that some varieties remain constant and others changing (recruited and lost in different cycles), we conducted interviews about the reasons why households consider replaceable or not a traditional variety and how the not replaceable variants are maintained and recovered.

#### Technological factors

Interviews were conducted to obtain information on: (i) tools and practices representing traditional agriculture, including traditional techniques and mutual help, (ii) land area cultivated with native tubers, and (iii) strategies for maintaining seed flow among households, including data on effort invested in searching seeds, and mechanisms of seeds attainment. With this information we constructed an index of traditional agricultural management (ITAM, Tables 1 and 2).

#### Socio-economic factors

The following socio-economic factors were analyzed: type of land tenure, self-sufficiency, family size, and family hand labor. An index of self-sufficiency (ISS) was constructed to analyze the relation between the monetary value of production destined to direct consumption and incomes earn by households. The index of family size (IFS) was designed to evaluate the relation between number of members of households and the maximum number of members in households. The index of hand labor (IHL) analyzed the relation between number of households members older than 12 years old (age at which a member of a household is locally considered to be a worker) and the maximum number of members with this age in households.

### Data analyses of influencing factors

Linear regressions between traditional varieties richness of native tubers as dependent variable of 10 independent variables (Table 2) were performed per region and per agricultural cycle. Multivariate statistical analyses (cluster and principal components analyses [41]) were performed through SPSS 9.0 [42] to determine similarities of households according to all the variables documented in the study (Table 3). For principal components analysis a correlation matrix was calculated using the Pearson's coefficient and variables standardized. Eigenvectors were calculated to identify factors with higher weight in the principal components. These analyses along with linear regressions allowed exploring from different perspectives meaningful factors influencing management of richness of traditional farmer varieties.

**Table 3:** Data matrix of the variables and indexes used in cluster and principal components analyses

Variables	Abanto P	Abanto S	Huaccha	Carrera	Rojas	Cabrera	Rosado	Alejo	Aquino	Fernández	Sánchez	Antonio
	
	C1	C2	C3	C4	C5	C6	H1	H2	H3	H4	H5	H6
Potato varieties richnes	66	133	289	67	57	52	139	154	57	252	111	203

Oca varieties richnes	13	16	19	16	17	10	54	41	24	56	28	59

Olluco varieties richnes	7	12	14	14	8	6	18	13	15	25	15	16

Mashua varieties richnes	5	7	9	6	3	3	55	19	14	45	13	35

Change of environmental conditions^a^	2.75	1.5	3.5	3.75	3.5	3.63	3.63	3.75	3.75	3.63	3.88	3.75

Farm zonification^c^	1	1	1	4	5	1	3	3	3	3	2	1

Number of farms	2	2	1	3	2	1	1	1	1	1	2	2

Farm area	4.5	5	15	12	5	3.5	6	50	8	108	28	12

Total number of plots	5	5	17	7	5	4	8	9	8	6	9	6

Land area cultivated with native tubers	1.75	1.4	6.37	1.33	1.94	3.2	4.5	6.5	3.87	8	6.02	5.55

Number of plots in the low zone	0	0	0	3	5	0	0	0	0	0	0	0

Number of plots in the middle zone	0	0	0	4	0	0	8	9	8	6	5	0

Number of plots in the high zone	5	5	17	0	0	4	0	0	0	0	4	6

Household head language^d^	2	2	2	2	2	2	1	1	1	1	1	1

Household head age learning agriculture^e^	1	1	1	1	0.5	0.25	1	1	1	1	1	1

Number of varieties received from parents	9	35	15	35	5	35	90	50	30	50	50	250

Origin of cultivated land^f^	1	0.25	1	0.5	1	1	1	0.5	0.75	0.75	1	1

Household head age	33	41	48	56	57	36	41	55	56	50	39	29

Age as independent farmer	25	23	17	23	25	24	22	18	24	22	18	12

Number of agricultural traditions practiced	0	1	3	2	1	2	1	5	2	2	6	3

Enhancing children to practice agriculture	2	0	2	0.67	0	1.33	2.33	3	3	3	3	1

Fallow period of land (years)	5	4	4	6	3	6	6	3	3	6	3	6

Tilling with chakitaqlla^g^	0	0	0	0	0	0	1	1	0.5	0.5	0.5	1

Use of not commercial inputs in agriculture	0.47	0.61	0.5	0.75	1	1	1	1	1	0.67	1	0.57

Traditional seed storage^h^	0	1	1	0	1	1	0	1	0	0	0	1

Mutual help^i^	3	2	2	2	2	2	2	2	1	2	2	2

Seed search effort	49	48	66	66	20	24	63	0	12	66	12	49

Seed getting mechanisms^j^	1	0.4	0.4	1	0.8	0.6	0.6	0	0.8	0.4	1	0.4

Household head's provenance^k^	2	2	2	2	1	2	1	2	1	1	1	1

Number of native parents, grandparents, and great-grandparents	0	0	0	0	6	3	1	0	3	2	4	2

Willingness of staying in the community	3	4	3	4	4	1	3	4	4	4	1	3

Territorial vision^l^	2	2	1	1	2	1	2	1	3	3	1	1

Migration frequency of household head	1	0	1.5	0.4	0	0	0	0	0	0	0	0

Migration of other family members	0	0	0.6	0	0.17	0	0.13	0.5	0.56	0.86	0	0

Monetary value of production directed to self-consumption	316.91	1,780.00	12,736.40	95.2	286	2,951.75	5,395.00	5,300.00	2,699.30	22,255.50	6,750.00	11,671.25

Income from production selling	4,809.65	6,132.00	16,171.60	3,221.80	2,184.00	6,186.75	8,555.00	4,325.00	369.6	13,055.00	1,904.00	5,860.00

Income from hand labor selling	0	0	0	0	0	0	787.5	0	0	0	1,344.00	0

Income from governmental subsidies	0	0	0	0	0	0	1,200.00	1,200.00	1,200.00	1,200.00	1,200.00	1,200.00

Total number of family members	4	6	11	9	7	4	10	8	10	8	4	5

Family members at labor age	2	6	7	4	4	2	5	5	6	5	2	2

Number of held family members	4	6	10	6	7	4	9	5	6	6	4	5

Number of men composing the family	3	4	7	3	3	2	6	3	3	1	1	1

Number of women composing the family	1	2	3	3	4	2	3	2	3	5	3	4

^a ^1 to < 2 = favorable; 2 to < 3 = without change; 3 to < 4 = unfavorable;^**b **^1 = private; 2 = communal;^**c **^1 = high zone; 2 = middle and high zones; 3 = middle zone; 4 = middle and low zones; 5 = low zone;^**d **^1 = Quechua; 2 = Quechua Spanish;^**e **^1 = child; 0.5 = adolescent; 0.25 = adult;^**f **^1 = heritage; 0.75 = apportioning; 0.5 = rent/sharing; 0.25 = purchase;^**g **^1 = raway; 0,5 = pampay; 0 = furrow;^**h **^1 = coyona, saway; 0 = platforms, nets, sacks;^**i **^1 = only mutual help; 2 = mutual help and farm workers; 3 = reduced mutual help and farm workers;^**j **^1 = barter and gift; 0.8 = barter or gift; 0.6 = barter, gift and purchase; 0.4 = purchase and barter or gift; 0.2 = purchase; 0 = anyone;^**k **^1 = native; 2 = foreign;^**l **^1 = limited for study; 2 = limited for work; 3 = limited for study and work.

## Results

### Richness of traditional varieties of native tubers and their wild relatives

**Figure 3:**
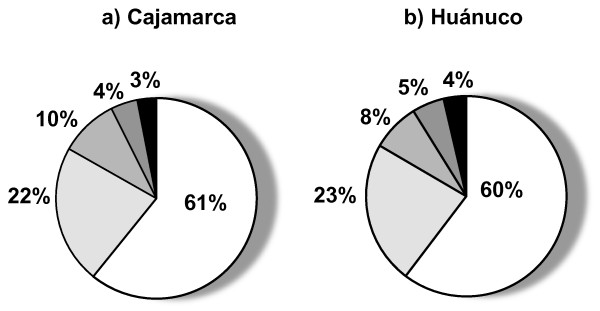
**Distribution of native traditional varieties of potato among farmers**. a) Cajamarca b) Huánuco. Production cycles 2001-2005. Categories: very rare, rare, roughly common, common, very common.

**Figure 4:**
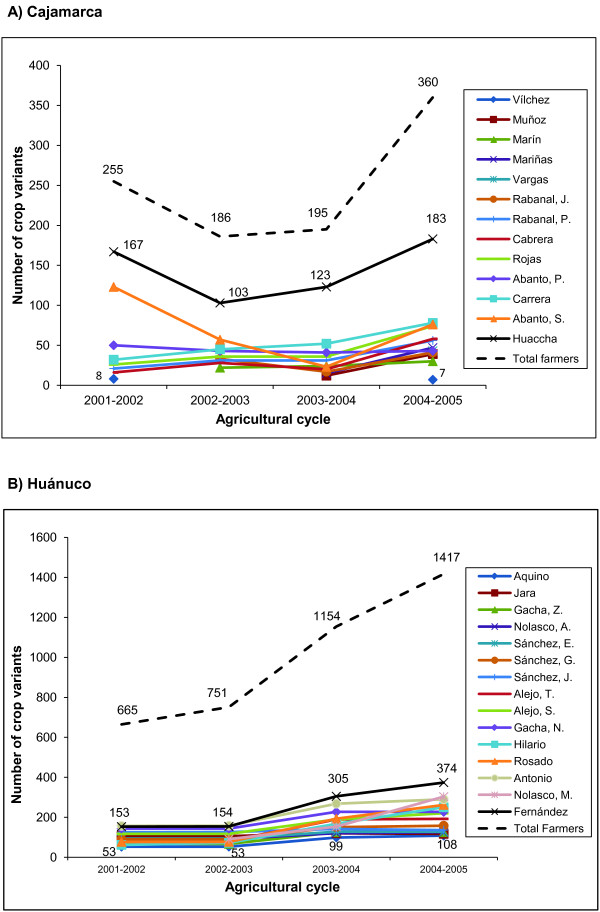
**Annual variation of the traditional varieties of native tubers richness from farmers**. A) Cajamarca B) Huánuco. Period 2001-2005.

Our study documented a high number of traditional farmer varieties of native tubers among the households studied. At regional level, richness of traditional varieties was markedly greater in Huánuco (1483 names of traditional farmer varieties) than in Cajamarca (507 names of traditional farmer varieties), predominating the traditional farmer varieties of potato in both richness and cultivated area (Table 4). We found significantly more varieties managed per household per year in Huánuco (146.39 ± 12.02) than in Cajamarca (44.55 ± 9.26) (mean ± S.E., F = 42.949, *p *< 0.0001), and marked differences in number of varieties per year among households within each region (on average 78.25 to 246.50 in Huánuco, 7.50 to 144.00 in Cajamarca) (Table 5). Also, we recorded marked asymmetry in number of traditional farmer varieties managed by households. In Cajamarca Mr. Huaccha accounted 65% of the regional traditional farmer varieties and Mr. Vílchez had only 2%, whereas in Huánuco Mr. Fernández had 26% of the regional traditional farmer varieties and Mr. Aquino only 7% (Tables 4 and 5). In both regions a high number of traditional farmer varieties are "very rare" found only in single households (546 in Huánuco, 239 in Cajamarca). Figure 3 describes this pattern for the traditional farmer varieties of potato. Despite the loss of traditional varieties in some communities of Cajamarca, a continuous trend of increasing traditional farmer varieties richness was found during the period studied in both regions and in all households monitored (Figure 4).

**Table 4:** Traditional farmer varieties richness of native tubers from Cajamarca and Huánuco during four agricultural cycles (2001-2005)

Region/Farmer	Farmer varieties richness				Total varieties richness	Percentage of regional varieties richness
			
	Potato	Oca	Olluco	Mashua		
*CAJAMARCA*						

HUACCHA	289	19	14	9	331	65.3

ABANTO, S.	133	16	12	7	168	33.1

CARRERA	67	16	14	6	103	20.3

ABANTO, P.	66	13	7	5	91	17.9

ROJAS	57	17	8	3	85	16.8

CABRERA	52	10	6	3	71	14.0

RABANAL A., P.	44	12	6	3	65	12.8

RABANAL V., J.	44	5	2	3	54	10.7

VARGAS	28	20	2	2	52	10.3

MARIÑAS	32	9	3	3	47	9.3

MUÑOZ	32	4	2	1	39	7.7

MARIN	26	8	2	3	39	7.7

VÍLCHEZ	12				12	2.4

**Total different varieties**	**402**	**62**	**27**	**16**	**507**	**100.0**

*HUÁNUCO*						

FERNÁNDEZ	252	56	25	45	378	25.5

NOLASCO, M.	192	53	40	48	333	22.5

ANTONIO	203	59	16	35	313	21.1

ROSADO	139	54	18	55	266	17.9

HILARIO	177	31	29	17	254	17.1

GACHA, N.	160	27	19	28	234	15.8

ALEJO, S.	154	41	13	19	227	15.3

ALEJO, T.	139	42	12	16	209	14.1

SÁNCHEZ C., J.	138	15	15	6	174	11.7

SÁNCHEZ S., G.	111	28	15	13	167	11.3

SÁNCHEZ V, E.	128	13	11	13	165	11.1

NOLASCO, A.	125	12	7	6	150	10.1

JARA, E.	108	14	4	3	129	8.7

GACHA, Z.	77	28	10	14	129	8.7

AQUINO	57	24	15	14	110	7.4

**Total different varieties**	**910**	**240**	**141**	**192**	**1483**	**100.0**

**Table 5:** Traditional farmer varieties richness of tubers per agricultural cycle, with number of fields and cultivated area, per household

Region	Farmer	Number of farmer varieties of tubers				Annual average of richness	Number of fields	Annual average cultivated area
					
		Cycle 1	Cycle 2	Cycle 3	Cycle 4			
Cajamarca	Huaccha	167	103	123	183	144.00	17	1.59
	
	Abanto S	123	57	23	76	69.75	5	0.35
	
	Carrera	32	45	52	78	51.75	7	0.33
	
	Abanto P	50	43	41	43	44.25	5	0.44
	
	Rojas	26	36	36	74	43.00	5	0.49
	
	Vargas		40		43	41.50	2	0.02
	
	Rabanal P	21	31	31	56	34.75	6	1.44
	
	Mariñas			15	47	31.00	3	0.55
	
	Cabrera	16	28	20	58	30.50	4	0.80
	
	Rabanal J		33	17	41	30.33	3	0.16
	
	Muñoz			12	39	25.50	2	0.36
	
	Marín		22	24	30	25.33	3	0.33
	
	Vilchez	8			7	7.50	2	0.01

Huánuco	Fernández	153	154	305	374	246.50	6	2
	
	Antonio	157	158	268	290	218.25	6	1.39
	
	Gacha N	144	144	227	227	185.50	9	1.53
	
	Nolasco M		91	153	303	182.33	3	1.58
	
	Alejo S	117	117	189	220	160.75	9	1.63
	
	Alejo T		81	189	192	154.00	3	0.84
	
	Rosado	77	77	193	262	152.25	8	1.13
	
	Hilario	62	72	167	250	137.75	6	1.63
	
	Sánchez J	125	126	142	136	132.25	7	1.22
	
	Sánchez G	90	90	151	158	122.25	9	1.51
	
	Jara	106	106	120	117	112.25	8	0.9
	
	Sánchez E	93	93	128	128	110.50	5	0.9
	
	Nolasco A	91	91	121	125	107.00	7	1.55
	
	Gacha Z	67	67	125	125	96.00	6	1.41
	
	Aquino	53	53	99	108	78.25	8	0.97

A total of 10 plant species were recorded and documented as wild relatives of potato, oca, olluco, and mashua in Cajamarca (5 were recognized by local people using the term ***sacha ***meaning wild in Quechua, e. g. "***sacha papa***", see Table 6), whereas 6 species of potato and oca were recorded in Huánuco (8 were recognized by local people, including wild relatives of olluco and mashua). The crop wild relatives identified are used by people for different purposes (Table 6). One farmer said that sometimes cultivated plants breed with their corresponding ***sacha***, and it is possible to find in wild populations varieties showing mixed features of both cultivated and wild plants. He added that from time to time some of these plants are adopted for cultivation as new varieties.

**Table 6:** Wild relatives of native tubers identified, their local names and information on their management and use.

Scientific name	Peasant name	Management and type of use
*CAJAMARCA (Cuenca de Muyoc)*		

*Solanum jalcae* *Solanum chomatophylum* *Solanum chiquidenum*	"papa de zorro" "sacha papa"	Management: conservation of the "ambulco" (bulb) protecting it from the animals. Type of use: food; seed; sale.

*Oxalis spp*.(5 species not identified)	"oca de zorro" "sacha oca"	Type of use: health; food; seed; sale.

*Ullucus aborigineus*	"olluco de zorro" "sacha olluco"	Management: protecting from animal's consumption. Type of use: health; food; seed; sale.

*Tropaeolum sp*.	"mashua de zorro" "maca de los gentiles" "sacha mashua"	

*HUANUCO (Cuenca de Mito)*		

*Solanum dolichocremastrum *ex *Solanum chavinense* *Solanum ambosinum* *Solanum bucasovi* *Solanum huanucense*	"jupay papa "pishgush papan" "sacha papa"	Type of use: health.

*Oxalis san-miguelii *Knuth *Oxalis huanuquense*	"ogausho" "purun chulco" "sacha oca"	Type of use: health; food

No fueron colectadas especies	"jupay llutu" "jupay olluco" "sacha olluco"	. Type of use: health

No fueron colectadas especies	"jupay mashua" "purun mashua" "sacha mashua"	Type of use: health

### Factors influencing maintenance of native tubers variation

#### Environmental conditions

According to farmers' perception, the most severe pests threatening agriculture in Cajamarca and Huánuco are the potato blanch or "*rancha*" (*Phytophthora infestans*) and the Andean potato weevil or "*gorgojo de los Andes*" (*Premnotrypes *spp.). Frosts are also considered a principal factor affecting agriculture. In addition, the farmers interviewed said that delay in starting of the rainy season contribute to decrease ponds and soil humidity and addressed problems of soil erosion and decreasing fertility.

According to farmers' perception, distribution and abundance of wild relatives are decreasing in great part of their territories. They consider that this situation is mainly due to damage caused by skunks which unbury tubers looking for insect larvae for feeding; also, because of over-grazing of domestic animals, accidental and intentional burning of vegetation, and drought. Some people also referred to decreasing of vegetation cover caused by deforestation and land use change, especially because of agriculture expansion.

In order to face environmental degradation, farmers are trying to recover traditional strategies and practices, like rotation and lying at rest of soils, production of livestock manure in particular areas (***potreros***, ***majadeos***, ***guaneos***), grazing land management, regulating slash and burn agricultural systems (***rozo y quema***) and grassland burning practices. They are adopting appropriate technologies of conservation of soil (e.g. terraces, protection fences, and organic fertilization), water (e.g. water reservoirs, optimization of irrigation, watershed management) and vegetation cover, mainly through management of agroforestry systems. The perspective of these measures is to guarantee an adequate habitat for native tuber crops, their varieties and wild relatives.

#### Organization of cultivated land area

Clear differences among regions and households were observed in land area dedicated to cultivate native tubers and in number and altitudinal distribution of parcels (Table 7). Correlation between varieties richness and cultivated area were significant in both regions (Table 2), and farm area was one of the most meaningful variables in multivariate analyses (Table 8). Households of Huánuco cultivated more land (3.9 to 8 ha) than those of Cajamarca (1.3 to 3.2 ha, with the exception of Huaccha, who cultivated 6.4 ha and 17 parcels). In Cajamarca, correlations between crop variation and the index of altitudinal distribution (IAD) and the total number of parcels were significant for each (R = 0.888, p = 0.003) and all agricultural cycles (R = 0.840, p = 0.006). In Huánuco households cultivated 6 to 9 parcels, whereas in Cajamarca households cultivated 4 to 7 parcels. In both regions parcels are distributed in high, intermediate and low zones, and agriculture of native tubers is mainly practiced in the higher zones. Summarizing, this information indicates that farmers using more cultivated land (in both regions), and more parcels in more altitudinal zones (this latter factor in Cajamarca), manage a greater richness of traditional varieties of tubers.

**Table 7:** Values of variables and indexes analyzed as factors influencing the richness of farmer varieties of tuber species

FARMER		Total number of farms	Total extent of the farm (ha)	Number of plots cultivated per farm	Area of land cultivated with native tubers 2001-2005 (ha)	Index of Parcel Distribution	Index of Cultural Identity		Index of Traditional Agricultural Management		Index of Self-Sufficiency		Index of Family Size		Index of Household Labor Hand	
						
						IPD	ICI	Level	ITAM	Level	ISS	Level	IFS	Level	ILH	Level
*CAJAMARCA*																

Alimarca	ABANTO P	2	4,5	5	1,8	0.29	0.18	Very weak	0.44	Middle	0.06	Very low	0.36	Middle	0.36	Small

Alimarca	ABANTO S	2	5,0	5	1,4	0.29	0.21	Weak	0.45	Middle	0.22	Low	0.55	Numerous	0.55	Middle

Trascorral	HUACCHA	1	15,0	17	6,4	1.00	0.43	Middle	0.63	Strong	0,44	Middle	1.00	Very numerous	1.00	Very big

Patiñico	CARRERA	3	12,0	7	1,3	0.26	0.29	Weak	0.53	Middle	0.03	Very low	0.82	Very numerous	0.64	Big

Rambrán	ROJAS	2	5,0	5	1,9	0.13	0.2	Weak	0.48	Middle	0.12	Very low	0.64	Numerous	0.64	Big

Carbón Alto	CABRERA	1	3,5	4	3,2	0.24	0.29	Weak	0.53	Middle	0.32	Low	0.36	Middle	0.36	Small

*HUÁNUCO*																

Monte Azul	ROSADO	1	6.3	8	4,5	0.37	0.4	Middle	0.65	Strong	0,34	Low	0.91	Very numerous	0.91	Very big

Monte Azul	ALEJO	1	50	9	6,5	0.41	0.69	Strong	0.55	Middle	0.49	Middle	0.73	Numerous	0.73	Big

Monte Azul	AQUINO	1	8	8	3,9	0.37	0.46	Middle	0.62	Strong	0.63	High	0.91	Very numerous	0.91	Very big

Monte Azul	FERNÁNDEZ	1	108	6	8,0	0.27	0.49	Middle	0.69	Strong	0.61	High	0.73	Numerous	0.73	Big

Huayllacayán	SÁNCHEZ	2	21	9	6,0	0.46	0.77	Strong	0.59	Middle	0.6	High	0.36	Middle	0.36	Small

San Juan de Tingo	ANTONIO	2	8	6	5,6	0.35	0.62	Strong	0.66	Strong	0.62	High	0.45	Middle	0.45	Middle

**Table 8:** Eigenvectors of the most meaningful variables in the first three principal components.

Variable	Component 1	Component 2	Component 3
Oca varieties richness	**0.92**	0.00	0.33

Mashua varieties richness	**0.92**	0.12	0.27

Total farm area	0.24	0.03	**0.93**

Language of the household head	**-0.83**	0.02	-0.27

Tilling using chakitaqlla	**0.92**	0.01	0.02

Migration of household's members	0.10	0.57	**0.72**

Monetary value of production directed to self-consumption	0.43	0.04	**0.75**

Incomes from governmental subsidies	**0.83**	-0.02	0.27

Total number of household's members	0.16	**0.96**	0.08

Households members at labor age	-0.02	**0.91**	0.18

#### Cultural identity and traditional agricultural management

Correlation between varieties richness and the index of cultural identity (ICI) was significant in both regions (Table 2). All farmers participating in our study are Quechua. All of them are part of family lineages that have practiced cultivation of tubers for several generations, and this practice constitutes the basis of their cultural identity. Therefore, farmers with greater cultural identity are those: i) Using the Quechua language, ii) maintaining more cultural traditions associated to agricultural activities such as ***yanamanchi ***or ***yanapanacushun ***(mutual help), agricultural rituals and festivities such as ***allpa pagamanchi ***or ***alpata garapashun ***(a ritual for feeding to the mother Earth or ***pacha mama***), the ***jircata garaicushun ***or ***cerrugpag ofrendapag ***(offering to the mountain), and agricultural calendar such as the ***quillata ***or ***goillarta***, or ***observaman ***(astronomic "reading") or ***señalcuna plantacuna ***and ***animalcuna mañucusheque climangpag ***(use of plants and animals as signals for climate prediction), iii) age of learning native tubers agriculture (younger: 12-18 years old), iv) stimulating and teaching children agricultural practices, v) management of a greater variation of native tubers (Table 7). Our study found that farmers from Huánuco have more characteristics of Andean traditional culture than those from Cajamarca.

**Figure 5:**
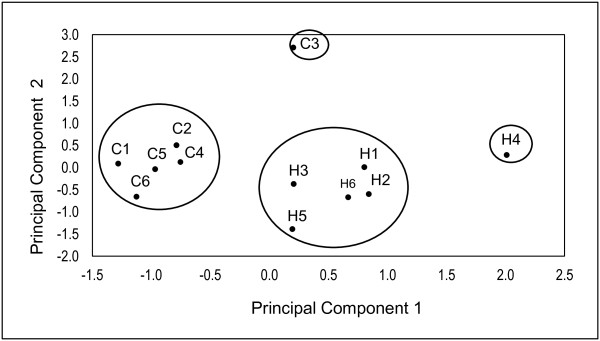
**Principal Components Analysis of the management of traditional varieties variation of native tubers among traditional farmers**. Cajamarca and Huánuco. The farmers with the prefix C are from Cajamarca and those with the prefix H are from Huánuco.

We also found that farmers from Huánuco practice agriculture more traditionally than those from Cajamarca (Table 7). The significant correlation between farmer varieties richness and the index of traditional agricultural management (ITAM) in both regions for all productive cycles (R = 0.540, p = 0.004) revealed the management of a high proportion of cultivated land with native tubers, the permanence of traditional agricultural techniques (long fallow period, tilling with ***chakitaqlla***, not using chemical inputs, and practicing traditional seed storing), mutual help (***yanamanchi ***or ***ayni***), and seed interchange (***rukan semilla***), as important characteristics for conserving farmer varieties richness (Table 3). The multivariate analyses identified that using of Quechua language, tilling with ***chakitaqlla***, and richness of oca and mashua managed were among the most meaningful factors differentiating farmers within and among regions (Table 8 Figure 5).

#### Self-sufficiency

The index of self-sufficiency (ISS) allowed identifying that in Huánuco most farmers participating in the study managed genetic resources orienting their production systems to direct consumption of products more clearly than farmers from Cajamarca (Table 7). The highest level of self-sufficiency was recorded in farmers who destined more products to direct consumption by their households. Intermediate levels were recorded in those who proportionally balance their production for commercialization and direct consumption. Low levels were recorded in households that destined most of their production to commercialization, and the lowest level in those who destined almost all their production to commercialization. Correlation between self-sufficiency and traditional varieties richness was significant in both regions in all the agricultural cycles studied (R = 0.283, p = 0.044), self-sufficiency economy favoring a greater number of farmer varieties.

#### Family size and hand labor

Differences in family size were found among the households studied. With the exception of younger families, most households cover their requirements of hand labor by their own family members (Table 7). Significant correlation of traditional varieties richness with the index of family size (IFS) and with the index of household hand labor (IHL) was identified only in Cajamarca for all the agricultural cycles (R = 0.658, p = 0.031; R = 0.670, p = 0.029, respectively), showing that in this region families with more members and hand labor manage more traditional varieties.

### Peasant strategies patterns for the *in situ *conservation of genetic resources

Cluster and principal components analyses clearly showed two groups of households which represent general patterns of strategies for *in situ *management of traditional varieties richness associated to the regions analyzed (Figure 5). The most meaningful factors classifying farmers in the first principal component were richness of oca and mashua, tilling with chakitaqlla, using Quechua and receiving governmental subsidies, all factors with higher levels in Huánuco than in Cajamarca (Tables 3 and 8). In the second principal component the total number of members and members contributing with hand labor to household were the most important. In the third principal component the amount of land cultivated, migration of household's members and amount of production directed to self-consumption were the most important (Table 8). Households from Huánuco manage greater traditional varieties richness, larger parcels and land area cultivated with native tubers, clearer signs of indigenous culture (mainly expressed in the use of the Quechua language, teaching of cultivation of native tubers and use of ***chakitaqlla***) and self-sufficiency. In Cajamarca, households' management strategy is mainly influenced by number and altitudinal distribution of parcels, family size and hand labor.

These analyses allowed distinguishing the farmers managing more traditional varieties within each group: Mr. Huaccha (with 331 traditional varieties in the whole period analyzed) in Cajamarca and Mr. Fernández (with 378 traditional varieties in the whole period analyzed) in Huánuco. Common aspects in these farmers are intermediate levels of the index of cultural identity (ICI) and high values of the index of traditional agricultural management (ITAM). The cases of these farmers support the information that indigenous culture favors maintenance of greater genetic resources diversity.

### Reasons influencing permanence and change of traditional varieties

**Figure 6:**
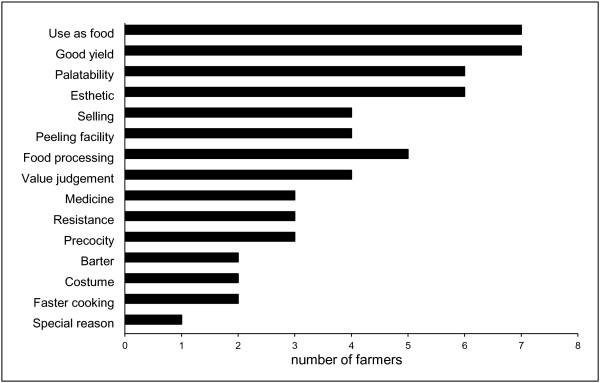
**Frequency of peasants reasons to cultivate different traditional varieties of native potatoes**. According to farmers testimonies from Cajamarca and Huánuco (2008).

Farmers' decisions for managing diverse traditional varieties of tubers take into account utilitarian, technical and cultural attributes of the varieties (Figure 6). The reasons more frequently mentioned by farmers for selecting and managing diversity of varieties were food quality and good productivity. Those traditional varieties that remained constant during all the productive cycles had the best attributes in these characteristics. Other attributes considered were form, color and pulp, as well as the properties for processing ***papa seca ***(dry potato) in Cajamarca and ***tocosh ***(a kind of fermented potato) and ***chuño ***(a frozen dehydrated potato) in Huánuco. Attributes for preparing these products are recognized specifically in some varieties, most of them remaining constant in seed stocks. Mr. Sánchez was the only one person addressing the conservation of traditional varieties diversity itself as the main reason for maintaining them.

## Discussion

Patterns of *in situ *conservation of agrobiodiversity documented in this study are carried out by households that are part of rural communities that maintain features of the classic Andean agriculture [5] such as: (i) management of a high variation of crops, (ii) vertical strategy of resources management with production systems adapted to specific ecological floors, looking for reducing environmental risk and optimizing use of diversity. Use and management of crop diversity, the pleasure associated to using such diversity, the prestige of managing it, and the security of resources availability to decrease risk are all expressions of that pre-modern agriculture.

According to Monroe [43], the capitalist modernization processes occurring in Peru during the last nearly six decades have not dissolved the traditional peasant community and its web of reciprocity relations. This assumption is generally correct; however, it is necessary to recognize that as our study documented, pre-modern expressions of agricultural management markedly vary among households and regions influenced by different factors associated to the incorporation of farmers into modern agriculture.

In general, we found that although the households studied maintain high diversity of traditional varieties of native tubers, some ecological and socio-cultural factors affect such task. Pests, climate change, and degradation of soils are all considered by farmers among the main unfavorable ecological factors limiting maintenance of traditional varieties diversity. In accordance with this perception, other studies [44,45] found that climate changes occurring from 2002 to 2004 determined loss of traditional varieties of native tubers in peasant communities of Cajamarca. During that period, atmospheric events affected the Andean region, decreasing precipitation, conditioning the presence of frosts (in Cajamarca frosts occurred in January caused loss of 70 to 100% of foliage coverage of potato, oca and olluco), and increasing the average annual temperature (for instance, in Cajamarca from 1 to 1.7°C) which favored the incidence of pests [45]. At regional level, ecological risk trends -decreasing of water supply, loss of vegetation cover, river pollution by mining and agrochemicals, soil erosion- are increasing, according to recent environmental diagnoses [46]. These trends threaten the resilience of production units and the *in situ *conservation of genetic resources. The risk can be even greater than what has been discussed in this study since most households in the villages studied conserve less variation than the households analyzed.

Our study found that the pattern of organization of the cultivated land area, characterized by using more parcels as possible in different altitudinal zones, favors the management of a greater variation of native tubers. Maintaining such actions is highly important for managing the current expressions of risk.

We also found that households with clearer signs of traditional Andean culture and traditional characteristics in agricultural management were those conserving more tuber varieties. Factors negatively affecting the indigenous Andean culture therefore represent general risks for conservation of traditional agricultural systems and agrobiodiversity. For instance, cultural discrimination influences changes in language, customs, food and use patterns of natural resources; migration and formal education may impact on young farmers' cultural identity and rooting to their communities [47-49], influencing cultural changes that may also modify processes involved in conserving variation of genetic resources. Actions against discrimination and for transforming the role of formal education into a way for reinforcing Andean culture are indispensable for the issue analyzed in this study.

The progressive loss of traditional techniques and agriculture intensification influence the loss of traditional farmer varieties diversity. A trend of expansion of the modern agriculture and livestock oriented predominantly to commercialization can be observed in both regions, and it may have unfavorable consequences for conservation of the genetic resources diversity. Trade of native traditional variaties is a controversial topic among actors promoting *in situ *conservation of agrobiodiversity. For some of them, markets determine high risk and frequently are direct cause of loss of native crops diversity since commercial systems generally favor a narrow spectrum of varieties [50], but also determine risk for the indigenous culture that model such diversity originally destined to a diversified rationality. According to Godoy [51], markets may affect patterns of natural resources use, social life aspects, and the indigenous people knowledge. However, after the Convention on Biological Diversity commercialization, particularly through fair markets, has been seen as a possible mechanism supporting biodiversity conservation, particularly because it can be an incentive for conserving native varieties through increasing their value, and because it may generate alternatives to destructive income-generating schemes [52]. However, this subject requires more studies, particularly in relation to cultural, economic and ecological consequences.

Our current information does not intend concluding linear cause-effect relations among the factors analyzed. Crop variation conservation is a complex issue influenced by socio-ecological factors like the ones analyzed in this study. Therefore, loss of variation associated to one particular factor may have consequences on other factors and on cultural patterns. This illustrates that partial examination of effects of environmental, cultural or socio-economic factors on crop variation offer insufficient information of the processes influencing *in situ *conservation of genetic resources. Holistic approaches and integrated analyses of these factors and processes are required.

Although our household sample was biased to conservationist farmers, the recorded ciphers of richness of traditional varieties of native tubers are encouraging signs of *in situ *conservation of Andean tuber crops occurring in the studied areas. However, some aspects also indicate vulnerability of the system; these are for instance the marked asymmetry in number of varieties managed by households (Table 2), as well as the high number of "very rare" varieties, found in single households (Figure 3). Enhancing the networks of seed exchange appears therefore to be a crucial aspect in policies aspiring to improve resilience of traditional agroecosystems and agrobiodiversity conservation and recovery.

The trend of increasing traditional variaties richness recorded during the period studied in both regions and in all households monitored is apparently related to actions of the "*in situ *project", which promoted use, cultivation and exchange of seeds, techniques and traditional knowledge of crop diversity. This pattern suggests that actions of conservationist farmers along with support of favorable external actors (e. g. NGOs, scholars, and governmental institutions) may be effective in accomplishing *in situ *conservation tasks, particularly if these efforts are sustained for long time periods as priority issue of public policies.

## Conclusions

Richness of traditional farmer varieties of native tubers was generally high in the conservationist households studied, but risks of varieties loss do exist associated to: a) unfavorable changes in ecological and socio-cultural contexts of their use and management, b) marked asymmetry in varieties richness managed by households, and c) a high number of unique varieties managed by single or few households.

Differences in traditional varieties richness managed by households were identified to be related to the extent of cultivated land area, degree of cultural identity, differences in the way of practicing traditional agricultural techniques, and levels of self-sufficiency of households. In Cajamarca differences were also related to altitudinal range of plots distribution, and family size.

Food quality, good productivity and culinary attributes, were main criteria considered by people for managing and selecting their tuber varieties, and influence decisions on which varieties are priority to remain constant.

The indexes designed in this study may contribute to more general analyses of the information generated by the "*in situ *project" on factors influencing peasant strategies of *in situ *conservation of genetic resources. Both quantitative and qualitative methods are required to conduct researches to support policies, actions and their monitoring for sustainable management of genetic resources. Proposals against genetic erosion risks require holistic research and action approaches for enhancing *in situ *conservation of crop variation and wild relatives.

Maintenance and promotion of indigenous Andean culture is crucial for ensuring maintenance of both traditional agroecological systems and agrobiodiversity. Policies supporting and respecting Andean culture through educational, cultural and economic programs are therefore directly connected with conservation of traditional farmer varieties. Promotion of seed availability and interchange are effective actions for maintaining and developing diversity, but using and valuing native tubers at regional, national and international levels are fundamental motivations to enhance processes in this direction.

Public policies are relevant for concerting visions to support planning and strategic actions oriented to the integral management of agrobiodiversity. In Peru, a favorable context currently exists for public policy in agrobiodiversity conservation issues. The Plan Nacional del Ambiente (National Environmental Plan) and the Programa Nacional de Agrobiodiversidad (National Program for Agrobiodiversity) are strategic instruments for recording and inventorying genetic resources, as well as identifying zones of agrobiodiversity at local, regional and national levels. Construction of public policies requires support from different sectors of the Peruvian society (farmers, NGO's, government, scientists, among others). Contributions in methods for recording, monitoring, systematizing and using field information are required. The experience of the "*in situ *project" is undoubtedly an important source of information and methodological experience. Our current analysis of a small sample of villages and farmers suggests particular pathways for systematizing and analyzing the valuable information generated by that project. But more formal reflections and actions from all the actors of the project are required for continuing constructing a vigorous program for conservation of plant genetic resources in one of the main centers of domestication of plants of the world.

## List of abbreviations

CCTA: Coordinadora de Ciencia y Tecnología en los Andes; CENTRO IDEAS: Centro de Investigación, Documentación, Educación, Asesoría y Servicios; CIECO: Centro de Investigaciones en Ecosistemas. Universidad Nacional Autónoma de México; CIP: Centro Internacional de la Papa; IDMA: Instituto de Desarrollo y Medio Ambiente; IN SITU PROJECT: In situ conservation of native crops and their wild relatives in Peru; INCAGRO: Programa para la Innovación y Competitividad del Agro Peruano; IIAP: Instituto de Investigación de la Amazonía Peruana; INEI: Instituto Nacional de Estadística e Informática; PNUD: Programa de las Naciones Unidas para el Desarrollo; PRATEC: Proyecto Andino de Tecnologías Campesinas; PROYECTO IN SITU: Proyecto Conservación In situ de Cultivos Nativos y sus Parientes Silvestres en el Perú; SENAMHI: Servicio Nacional de Meteorología e Hidrología; UNALM: Universidad Nacional Agraria La Molina.

## Competing interests

The authors declare that they have no competing interests.

## Authors' contributions

DVM conceived, designed and coordinated the study, carried out the analyses and helped to draft the manuscript. AC made substantial contributions to theoretical background, conception and design of the study, data analysis and interpretation of results, and drafted the manuscript. JTG participated in the conception and design of the study, analysis and interpretation of data and helped to draft the manuscript. ACS participated in the design of the study, carried out the statistical analyses and helped to draft the manuscript. All authors read and approved the final manuscript.

## Authors' information

DVM. MSc (Biology) at the Universidad Autónoma de México (UNAM), consultant of CCTA and researcher at the Centro de Investigaciones de Zonas Áridas, UNALM. Participant at the *in situ *project, combining ecological, ethnobiological, inter-cultutral and participatory research approaches.

AC. Biologist at UNAM, Mexico, PhD at the University of Reading, UK. Researcher at the Centro de Investigaciones en Ecosistemas, UNAM. Coordinator of the Laboratory of Ecology and Evolution of Plant Resources, advising and conducting researches with ethnoecological, ecological and evolutionary approaches to the study of plant management and domestication.

JTG. Lecturer at the Faculty of Biology, UNALM, Researcher and Director of the Centro de Investigaciones de Zonas Áridas, UNALM, and consultant of CCTA. Coordinator of researches in ecology of arid zones and mountainous ecosystems, and ethnobiology in relation to plant genetic resources.

ACS. Specialist in ecological economy, researcher of the Centro de Investigaciones de Zonas Áridas, UNALM, and consultant of CCTA. Participant at the *in situ *project, encharged of managing and analyzing databases of the project.
